# Testing the drivers of environmental persistence in bacterial pathogens

**DOI:** 10.1099/mic.0.001713

**Published:** 2026-06-08

**Authors:** Sarah Marde Mehdiabad, John J. Welch, Lucy A. Weinert, Camille Bonneaud

**Affiliations:** 1 Centre for Ecology and Conservation, University of Exeter, Penryn, Cornwall TR10 9FE, UK; 2 Department of Genetics, University of Cambridge, Cambridge CB2 3EH, UK; 3 Department of Veterinary Medicine, University of Cambridge, Cambridge CB3 0ES, UK; 4 Environment and Sustainability Institute, University of Exeter, Penryn, Cornwall TR10 9FE, UK

**Keywords:** abiotic conditions, bacteria, cell wall, ecology, environmental persistence, life history, transmission mode

## Abstract

Bacterial pathogens can increase transmission opportunities by surviving in the external environment, but despite wide variation in this ability, the drivers of such differences remain poorly understood. Here, we comparatively analysed data from 47 studies on 29 bacterial pathogens to investigate how phylogeny, structural traits (cell wall structure), life history (transmission mode, lifestyle, generation time) and abiotic conditions (temperature, humidity and surface material) influence persistence following deposition on inert surfaces. Our results showed that bacterial species differ consistently in persistence, but not in a way predicted by phylogeny. Of the bacterial traits, cell wall structure had a significant effect, with Gram-positive bacteria showing longer survival times. In contrast, transmission mode, lifestyle and generation time had no consistent effect on persistence. Bacteria also survived longer on inorganic surfaces, but with no significant effect of temperature or humidity. These findings indicate that both bacterial structural traits and abiotic conditions may play crucial roles in shaping bacterial persistence in the environment, likely through effects on resistance to hypoosmotic stress. How the benefits of persisting in dry external environments, for instance, by harbouring a Gram-positive cell wall, trade off with survival in hyperosmotic conditions or in the face of immune defences, however, remain to be determined.

## Data Availability

The data and R scripts reported in this paper can be accessed on the Microbiology Society Figshare [1]. These include bacterial species, cell wall structure (Gram-positive vs. Gram-negative), lifestyle (opportunistic vs. obligate), transmission mode (direct vs. indirect), surface type (organic vs. inorganic), humidity (%), temperature (°C), generation time (hours), survival (days), mortality on last measurement day (0/1). Data on taxonomic groupings are provided in Table S2.

## Introduction

Preventing and controlling infections by bacterial pathogens, including those that have acquired antimicrobial resistance, requires a solid understanding of their transmission. One key feature of bacterial transmission is the ability to persist and remain viable in the external environment [2,6]. Such an ability can increase opportunities for spread between hosts by increasing the likelihood of an encounter with a secondary host [7]. Despite critical implications for disease control, little is known about the environmental persistence of bacterial pathogens outside of nosocomial settings, and our understanding of the drivers of variation in environmental persistence remains incomplete.

The risk of infection through exposure to contaminated surfaces in healthcare settings has prompted most empirical studies of the environmental persistence of bacterial pathogens [8]. Such tests are typically performed by depositing known amounts of bacterial pathogen on air-drying surfaces and allowing them to desiccate for a given duration of time before measuring their survival as the ability to regrow on a plate or in liquid media [9]. For instance, following their deposition on glass surfaces, *Neisseria gonorrhoeae* was found to be capable of persisting for only 5 days [10], while *Acinetobacter baumannii* could persist for up to 329 days [11]. Such findings suggest that environmental persistence can vary significantly across bacterial species, although the precise drivers of this variation remain unclear.

Inter-specific differences in environmental persistence may be, at least partly, explained by differences in bacterial traits, such as cell wall structure and mode of transmission. Indeed, Gram-positive bacteria, which have a thicker, reinforced cell wall, are thought to be more resistant to environmental conditions and to desiccation than Gram-negative ones, because their cell wall provides greater protection from conditions, such as higher temperatures and decreased humidity [12]. For example, the Gram-positive bacterium, *Enterococcus faecium*, was found to be significantly more resistant to desiccation when deposited on anhydrous silica gel than the Gram-negative bacteria *Escherichia coli*, *Aeromonas hydrophila* and *Enterobacter cloacae* [12]. Furthermore, bacterial pathogens that favour indirect modes of transmission may persist longer in the environment than those that are directly transmitted. In support, *Salmonella enterica* serovar Enteritidis, which is transmitted indirectly via fomites, can persist in the environment for over 266 days [13], while the directly transmitted *N. gonorrhoeae* bacterium was found to persist in the environment for a maximum of 5 days only [10]. Although inter-specific variation in environmental persistence may in part be attributed to specific bacterial traits, tests of these associations remain challenging due to potentially confounding differences in experimental conditions across studies.

The duration of time that bacterial pathogens can persist in the environment is, indeed, also expected to differ as a function of the abiotic conditions experienced, such as temperature, humidity and the type of surface the bacteria are deposited on. Temperature should influence survival by changing or even disrupting vital cell components and processes, including compromising the integrity of DNA and RNA, affecting changes in the cell membrane and, at higher temperatures, denaturing proteins [14]. For example, *Listeria monocytogenes* was found to persist in water for 120 days at 4 °C, but only 50 days at 20 °C [15], while *E. coli* deposited on a wood surface survived for 28 days at 5 °C, but only 7 days at 20 °C [16]. Changes in humidity, on the other hand, may impact rates of dehydration and desiccation [17]. For instance, the persistence of *Acinetobacter baumannii* increased from 3 to 30 days when increasing the relative humidity of the environment from 10 to 31% [18]. Finally, surface type is also thought to affect environmental persistence, although the mechanisms by which this occurs vary across surfaces. For example, *Salmonella enterica* serovar Enteritidis deposited onto a copper surface survived for a shorter duration of time than those deposited on a steel or polymer surface, most likely due to the antimicrobial properties of the former [19,20]. *Streptococcus sanguis* deposited on porous surfaces persisted for a longer duration of time than those deposited on non-porous surfaces [21], most likely because surface unevenness may protect from environmental stressors [22]. Determining how abiotic factors affect persistence will not only improve our ability to predict differences in persistence across environmental conditions but will also clarify the role of bacterial traits in driving such variation.

Here, we conducted a test of the contribution of bacterial traits (phylogenetic, structural and life-history) and abiotic conditions in driving variation across measures of environmental persistence obtained following deposition on air-drying surfaces. To do so, we compiled environmental persistence data from the literature on 29 different bacterial pathogen species of humans and other species, which were obtained from laboratory studies under a range of experimental conditions. This allowed us to test the contribution of phylogeny, of structural (Gram-negative vs. Gram-positive cell wall structure) and life history (transmission mode, lifestyle and generation time) traits in the bacterial species, as well as of environmental conditions (temperature, humidity and surface material) in explaining variation in persistence following desiccation. We included transmission mode (direct vs. indirect) and lifestyle (opportunistic vs. obligate pathogen) because we might expect indirectly transmitting bacteria and those infecting hosts opportunistically to be able to persist for a longer duration of time in the environment than those that can only transmit directly or are fully reliant on hosts for survival. Furthermore, we included generation time because of the ubiquitous life history trade-off between replication rate and longevity [7,23]. Parsing out the role of bacterial and abiotic factors in driving differences in environmental persistence across bacterial pathogen species will help clarify links between persistence, virulence and transmission, thereby furthering our understanding of the role of environmental persistence in shaping bacterial transmission success.

## Methods

### Literature search

**Fig. 1.**
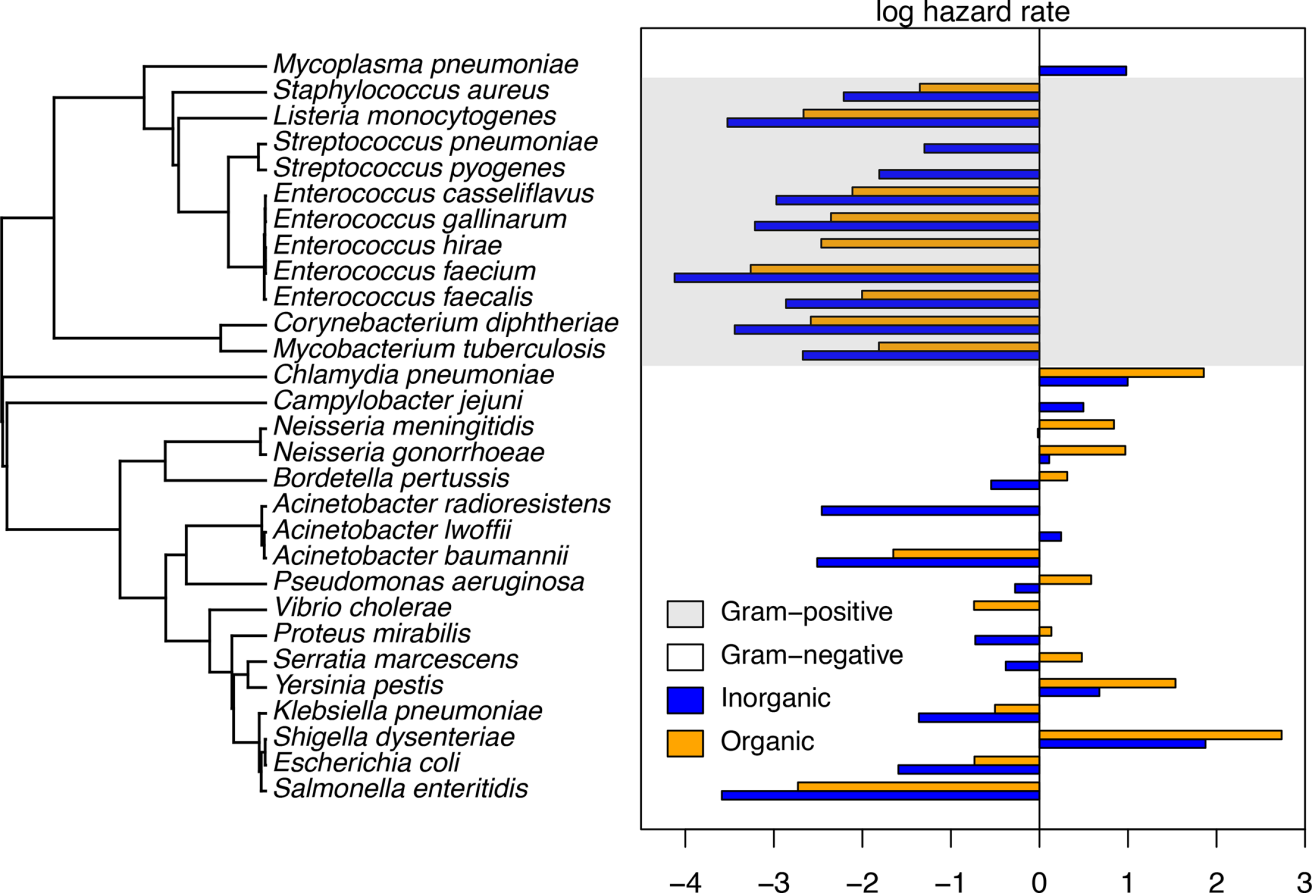
The bacterial species used in this study. The left-hand side shows the 29 bacterial species included here, alongside the phylogeny that was used to test for phylogenetic signal in the random ‘species’ effect (see Methods S1). The right-hand side illustrates the results of fitting a mixed-effect Cox proportional odds model to the survival data. Each bar is the mean over the fitted log hazard rates for each species on surfaces that were either inorganic (blue) or organic (orange). Lower log hazard rates imply longer survival times.

From September 2020 to August 2022, we searched the literature regularly using Google Scholar and by inputting various combinations of the following keywords: ‘bacteria’, ‘pathogen’, ‘environmental persistence’ and ‘survival’. We also searched the reference lists within the papers found. Papers were included when they met the following criteria: (1) they were published in a peer-reviewed academic journal; (2) they had measured environmental persistence for one or more bacterial species under one or more sets of laboratory conditions; (3) they provided information on the surface material used. Papers were screened and excluded following the PRISMA method (Fig. S1, available in the online Supplementary Material) [24]. Maximum environmental persistence was recorded as the last time point at which the pathogen was found viable, with viability determined as the ability to regrow after being deposited on an inert surface for a given amount of time. Details of excluded papers are provided in Table S1. For the bacterial species in our data set, we then compiled data on bacterial taxonomic grouping (phylum, class and order), as well as bacterial traits, namely cell wall type structure, lifestyle, transmission mode and generation time (Table S2). We defined as obligate, bacteria species that require a host to persist and replicate, and as opportunistic, those that can do so outside of a host. Because we were interested in testing whether bacterial pathogens capable of indirect transmission can persist longer in the environment than pathogens transmitting directly only, we classified the transmission mode of bacterial pathogens able to transmit both directly and indirectly as indirect. Finally, to test for phylogenetic effects, we generated a phylogeny of the species represented. Our tree was based on those of Hug *et al*. [25] and Dewar *et al*. [26] (see Methods S1) and is shown in Fig. 1.

We compiled environmental persistence data across a total of 47 studies of 29 distinct bacterial species (374 entries in total, with maximum environmental persistence data for a total of 269 entries). Roughly one-third of studies (*N*=14) measured the persistence of a single species under fixed environmental conditions. In contrast, 9 studies compared persistence across at least 2 species under identical environmental conditions (median=4 species compared, min=2, max=6), and 24 measured variation in the persistence of a single species across different environmental conditions. Overall, studies were conducted under a range of different environmental conditions, with temperature ranging from 4 to 27 °C (mean±se=23±1 °C), humidity from 9 to 90% (mean±se=37±3%) and 58 different surface materials. In our analyses, surface material was reduced to a minimum number of categories (organic vs inorganic) to reduce the probability of detecting type II errors (see Table S3). This will occur if, for example, a few species with longer environmental persistence are measured on a given surface type and insufficient numbers of species are included in each surface type. Reducing surface material to two categories also reduces errors in assigning surfaces to a specific type when details were lacking in the original study.

### Statistical analyses

To test for predictors of bacterial persistence time, we used a mixed-effect Cox proportional odds model, as implemented in the *coxme* package (v. 2.2–22; [27]) in R (v.4.4.4; [28]). Each data point comprised the last time of measurement (days) and a binary classification of whether mortality had occurred by that time. To explain variation in mortality probabilities, we fitted cell wall structure (Gram-negative vs. Gram-positive), transmission mode (direct vs. indirect), lifestyle (obligate vs. opportunistic) and surface material (organic vs. inorganic) as binary fixed effects. We fitted generation time (hours), temperature (°C) and humidity (%) as continuous fixed effects, and bacterial species as a random effect. We also allowed for possible effects of relatedness by treating the species-level phylogeny (Fig. 1) as a correlation matrix for these random effects [29]. All results of the model fitting are provided in Table S4. We note that an alternative, and in some ways preferable approach, would be to analyse each published study separately and then undertake a formal two-stage meta-analysis. However, we found too few studies that compared both Gram-negative and Gram-positive species, or inorganic and organic surfaces, to make this alternative possible (see Tables S5 and S6 for details).

## Results

Across 29 species of pathogenic bacteria (374 records in total), we tested whether species traits and abiotic factors predict their probabilities of environmental survival. An initial analysis-of-deviance showed that preferred models always included ‘bacterial species’ as a random effect, but without phylogenetic correlations between these effects (Table S4, ‘Tests for random effects’). This suggests that survival probabilities do differ consistently between species, but without a strong tendency for closely related species to be more similar. Table 1 shows results for the fixed factors under this preferred model. Results with the complete set of 374 records show only two significant predictors: cell wall structure and surface and similar results hold for the subset of 313 records where environmental temperature was measured. By contrast, results appear to change for the subset of 274 records with both temperature and humidity measurements. However, further investigation shows that two of the significant predictors are probably attributable to highly unbalanced representation (with 235/274 records being opportunistic pathogens with indirect transmission). As shown in Fig. S2, similar caveats apply to the apparently significant result for temperature. One final caveat is that tests of model adequacy suggest a poor fit, with residuals showing heterogeneity over time (see Table S4, ‘Model adequacy tests’). This makes the size of the effects more difficult to interpret; however, analyses of other subsets of the data (organic vs. inorganic surfaces and Gram-positive vs. Gram-negative species) show a better fit and yield the same patterns. Moreover, results for cell surface structure, which is strongly confounded with phylogeny (with only *Mycoplasma* being phenotypically Gram-negative but phylogenetically Gram-positive), are robust to the inclusion of a phylogenetic effect (see Table S4, ‘Best-fit coefficients’). Results were also robust to the inclusion of ‘study/species’ as a combined factor, attempting to control for heterogeneity between studies, as in a two-stage meta-analysis (see Table S4, ‘Best-fit coefficients’). As such, our data show two clear patterns: higher survival probabilities for Gram-positive species and higher survival on inorganic surfaces. Both effects are confirmed visually in Figs1  2, which summarize maximum survival times for the 269/374 records where mortality was observed.

**Table 1.** Probability of bacterial mortality outside the host, as a function of species traits and experimental conditions We report a mixed-effects Cox model, fitted to the day of last measurement, and the mortality outcome. Following model selection (Table S4, ‘Tests for random effects’), all results include ‘bacterial species’ as a random effect without phylogenetic correlations. Results are shown for the fixed effects; and for binary factors, coefficients apply to the first level listed (e.g. Gram-positive bacteria have a lower probability of mortality). Results are compared for the complete data set and for the subsets with usable temperature and humidity measurements.

	All data (=374)		With temp. (*n*=313)		With humidity (=274)	
Explanatory variable	Estimate (se)	(z) *P* value	Estimate (se)	(z) *P* value	Estimate (se)	(z) *P* value
Cell wall (Gram+ vs. –)	−2.19 (0.55)	(−3.98) <0.0001*	−2.69 (0.62)	(−4.35) <0.0001*	−1.87 (0.68)	(−2.77) 0.0056*
Transmission (indirect vs. direct)	−0.61 (0.70)	(−0.87) 0.39	−0.99 (0.79)	(−1.25) 0.25	−3.44 (1.05)	(−3.29) 0.0010*
Lifestyle (opportunistic vs. obligate)	−0.29 (0.71)	(−0.41) 0.68	0.13 (0.80)	(−0.16) 0.98	3.24 (1.24)	(2.61) 0.0091*
Generation time (hours)	0.00 (0.02)	(0.23) 0.82	0.01 (0.02)	(0.36) 0.73	0.09 (0.06)	(1.47) 0.14
Surface (organic vs. inorganic)	0.86 (0.15)	(5.90) <0.0001*	1.03 (0.17)	(6.17) <0.0001*	1.12 (0.18)	(6.27) <0.0001*
Temperature (°C)	–	–	0.04 (0.03)	(1.66) 0.10	−0.10 (0.04)	(−2.59) 0.0095*
Humidity (%)	–	–	–	–	0.00 (0.01)	(0.06) 0.95

**Fig. 2.**
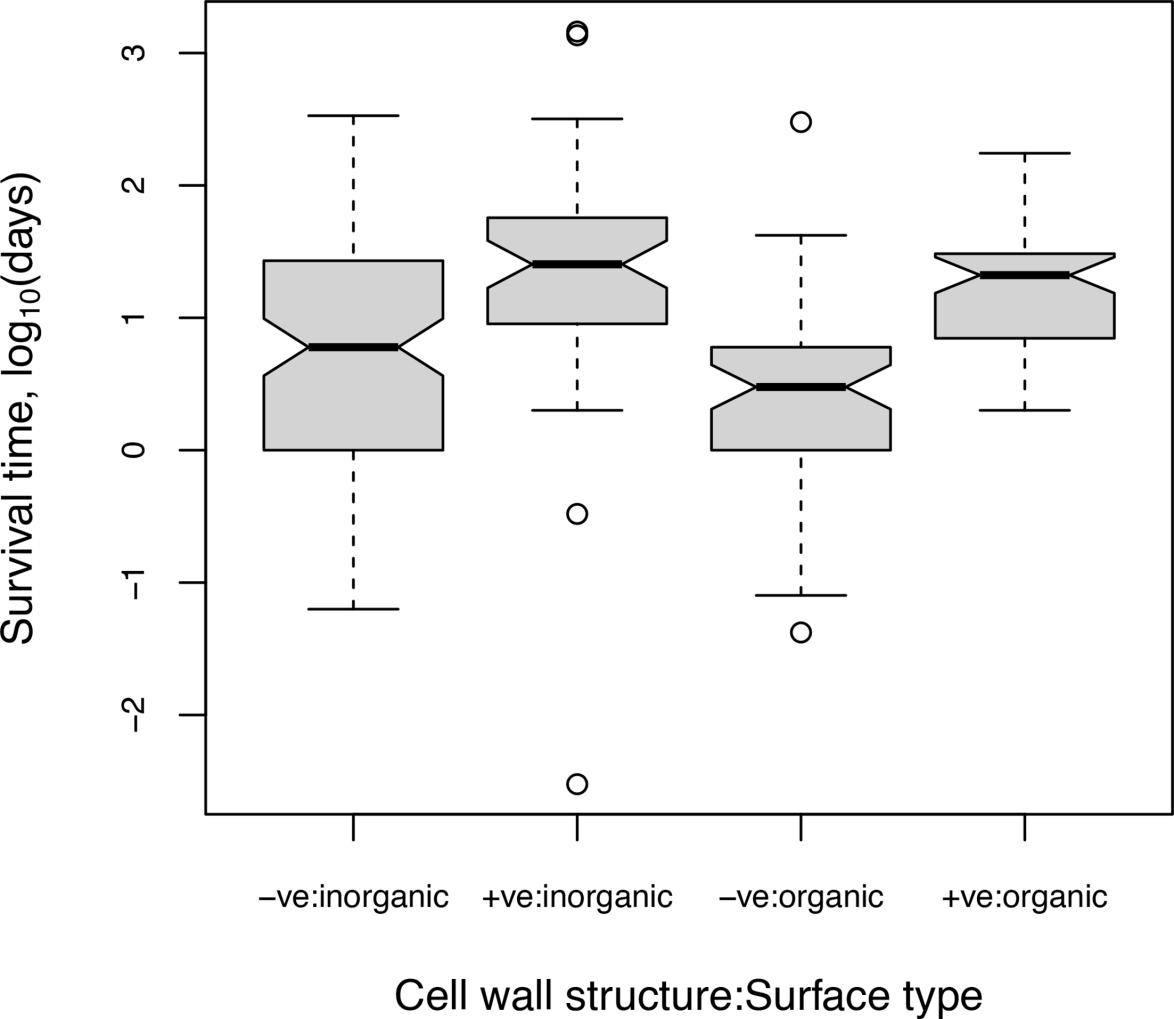
Survival probability is increased for Gram-positive species, and on inorganic surfaces. Boxplots show the maximum day of survival for the subset of our data (269/374 records), where mortality was observed during the experiment. Results suggest a clear and consistent trend for Gram-positive (+ve) species to survive longer than Gram-negative (-ve) species, and a weaker trend for longer survival on inorganic surfaces relative to organic ones. This is confirmed by the more formal analysis reported in Table 1.

## Discussion

Environmental persistence is likely to play a critical role in bacterial pathogen transmission, but our understanding of the factors that shape it and, specifically, the contribution of bacterial traits vs. abiotic conditions, is curtailed by a lack of tests conducted across a sufficient number of bacterial species and under varying experimental settings. Here, we make use of published data on environmental persistence across 29 bacterial species to clarify such associations. First, we found that measures of persistence obtained across studies were repeatable within species, with preferred models including ‘bacterial species’ as a random effect, though without any clear tendency for closely related species to have similar persistence times. Second, variation in persistence was significantly explained by bacterial traits and abiotic factors. Cell wall structure, but not transmission mode, bacterial lifestyle or generation time, was consistently associated with variation in environmental persistence. Furthermore, surface material was also significantly associated with persistence, with bacteria persisting longer when deposited on inorganic surfaces than on organic ones.

Bacterial cell walls maintain the structural integrity of cell shape [30], protect against lysis from high internal osmotic pressure [31] and constitute barriers that shield against external chemical stresses, viruses, other micro-organisms and host immune systems [32,35]. The strength and rigidity of the cell wall depend on the presence of peptidoglycans, which can be shaped either as a thick, multi-layered sheath on the outside of the plasma membrane in bacteria retaining the Gram stain (i.e. Gram-positive), or as a single layer in the periplasmic space between outer and inner membranes in Gram-negative ones [30]. Bacteria staining Gram-positive are, therefore, expected to be more resistant to desiccation than Gram-negative bacteria. In support, a comparison of the survival ability of Gram-positive (*Enterococcus faecium* and *Lactobacillus plantarum*) and Gram-negative (nine *Salmonella* strains, *Enterobacter cloacae*, *E. coli*, *Pseudomonas aeruginosa*, *Aeromonas hydrophila* and *Aeromonas sobria*) bacteria desiccated on anhydrous silica gel for >3 months showed a greater resistance of the former, with survival rates ranging from 30 to 100% for Gram-positive bacteria to 5% or less for Gram-negative ones [12]. Our analysis of persistence across 29 bacterial species shows concordant findings, with Gram-positive bacteria surviving for twice as long as Gram-negative ones after deposition on inert surfaces. While together, these results evidence the advantage of Gram-positive cell walls for persisting in situations of heightened turgor pressure (e.g. when the intracellular osmotic pressure is high due to being in a dry external environment), the costs of such a cell wall under other circumstances may give rise to trade-offs between environmental persistence and other life history traits.

There can, indeed, be costs associated with Gram-positive cell wall structures and benefits to harbouring a Gram-negative cell wall or even no cell wall at all [36,37]. The costs of a Gram-positive cell wall mainly stem from the thicker peptidoglycan layer, which increases vulnerability to cell-wall targeting antibiotics and to hyperosmotic stress conditions (when environmental fluid osmolarity is greater than within the cell), which are, for example, commonly experienced in soil [36]. The benefits of Gram-negative bacteria, on the other hand, include an outer membrane that can act as a barrier against antibiotics and chemical agents, as well as presents lipoproteins, such as lipopolysaccharide, that can stimulate the immune system and enable disease progression and transmission [38]. While some Gram-negative bacteria are permanently cell-wall deficient (i.e. the bacterial phylum *Mycoplasmatota*), others can transiently lose their cell wall under stress [36]. For example, Gram-negative *Pseudomonas aeruginosa* and *Vibrio cholerae* can temporarily shed their cell wall when exposed to *β*-lactam antibiotics to avoid bulging and cell death [39,40], and Gram-positive bacteria, such as *Streptomyces viridifaciens*, can produce cell-wall-deficient cells (l-forms) when exposed to cell-wall-targeting antibiotics and high levels of osmolytes in the media [41,42]. Trade-offs between the benefits of a Gram-positive cell wall for persisting in the external environment during transmission, and the advantages of a Gram-negative wall structure or even of being cell-wall deficient in the face of other environmental threats, raise the hypothesis that differences in cell wall structure are associated with different bacterial life history strategies.

Effects of the external environment on the internal osmotic pressure may also, in part, explain the association between surface type and persistence. For instance, deposition in water is expected to rapidly cause cell membranes to burst from osmotic stress [30], and *E. coli* was found to survive for longer durations of time on lettuce and bell peppers when these were placed under drier conditions [43]. Furthermore, more porous surfaces may allow bacteria to nestle into pores and reduce their exposure to ambient conditions, including changes in humidity levels [44,45]. Although our study does not allow us to identify the mechanisms mediating a link between surface type and environmental persistence, our finding that bacteria can survive for longer durations on inorganic versus organic surfaces is consistent with previous work. For example, methicillin-resistant *Staphylococcus aureus* was found to survive for 318 days on glass [11] and only 21 days on fabric [46]. Such a contrast suggests that built environments may enhance bacterial persistence outside of hosts, and that risks of bacterial transmission may be exacerbated in urban relative to natural environments.

In summary, our comparative analysis highlights the role of both biotic and abiotic factors in explaining variation in environmental persistence across bacterial species. A rigid Gram-positive cell wall structure that can withstand high osmotic pressure may be beneficial because, by enabling longer persistence in the environment, it increases the chances of transmission to secondary hosts. Nevertheless, a cell wall also entails costs, such that the benefit of increased environmental persistence on inert surfaces may come at the cost of persistence in soil, in the face of competition from other bacteria or of assaults from the host immune system. Further work is needed to resolve how bacterial traits correlate and trade off with each other and potentially give rise to different life history strategies.

## Supplementary material

10.1099/mic.0.001713 Supplementary Material 1.

10.1099/mic.0.001713 Supplementary Material 2.

## References

[1] Mehdiabad SM, Welch JJ, Weinert LA, Bonneaud C (2026). Testing the drivers of environmental persistence in bacterial pathogens. Figshare (Preprint).

[2] Bonhoeffer S, Lenski RE, Ebert D (1996). The curse of the pharaoh: the evolution of virulence in pathogens with long living propagules. Proc R Soc Lond B.

[3] Ewald PW, Barbosa PA, Schultz JC (1987). Insect Outbreaks.

[4] Ewald PW (1994). The Evolution of Infectious Disease.

[5] Ewald PW (1995). The evolution of virulence: a unifying link between parasitology and ecology. J Parasitol.

[6] Mitchell KM, Churcher TS, Garner TWJ, Fisher MC (2008). Persistence of the emerging pathogen *Batrachochytrium dendrobatidis* outside the amphibian host greatly increases the probability of host extinction. Proc Biol Sci.

[7] Walther BA, Ewald PW (2004). Pathogen survival in the external environment and the evolution of virulence. *Biol Rev Camb Philos Soc*.

[8] Kramer A, Schwebke I, Kampf G (2006). How long do nosocomial pathogens persist on inanimate surfaces? A systematic review. BMC Infect Dis.

[9] Wendt C, Dietze B, Dietz E, Rüden H (1997). Survival of *Acinetobacter baumannii* on dry surfaces. J Clin Microbiol.

[10] Elmros T (1977). Survival of *Neisseria gonorrhoeae* on surfaces. Acta Derm Venereol.

[11] Wagenvoort JHT, Sluijsmans W, Penders RJR (2000). Better environmental survival of outbreak vs. sporadic MRSA isolates. J Hosp Infect.

[12] Janning B, in’t Veld PH (1994). Susceptibility of bacterial strains to desiccation: a simple method to test their stability in microbiological reference materials. Analytica Chimica Acta.

[13] Davies RH, Wray C (1996). Persistence of *Salmonella enteritidis* in poultry units and poultry food. Br Poult Sci.

[14] Russell AD (2003). Lethal effects of heat on bacterial physiology and structure. Sci Prog.

[15] Budzinska K, Wronski G, Szejniuk B (2012). Survival time of bacteria *Listeria monocytogenes* in water environment and sewage. Polish J Environ Stud.

[16] Williams AP, Avery LM, Killham K, Jones DL (2005). Persistence of *Escherichia coli* O157 on farm surfaces under different environmental conditions. J Appl Microbiol.

[17] Lin KS, Marr LC (2020). Humidity-dependent decay of viruses, but not bacteria, in aerosols and droplets follows disinfection kinetics. Environ Sci Technol.

[18] Jawad A, Snelling AM, Heritage J, Hawkey PM (1998). Exceptional desiccation tolerance of *Acinetobacter radioresistens*. J Hosp Infect.

[19] Faúndez G, Troncoso M, Navarrete P, Figueroa G (2004). Antimicrobial activity of copper surfaces against suspensions of *Salmonella enterica* and *Campylobacter jejuni*. BMC Microbiol.

[20] Warnes SL, Keevil CW (2013). Inactivation of norovirus on dry copper alloy surfaces. PLoS One.

[21] Elftonson JE, Ström G, Holmberg K, Olsson J (1996). Adhesion of *Streptococcus sanguis* to porous and non-porous substrates with well-defined surface energies. J Adhesion Sci Technol.

[22] Montanaro L, Arciola CR (2000). Handbook of Bacterial Adhesion.

[23] Boonekamp JJ, Salomons M, Bouwhuis S, Dijkstra C, Verhulst S (2014). Reproductive effort accelerates actuarial senescence in wild birds: an experimental study. Ecol Lett.

[24] O’Dea RE, Lagisz M, Jennions MD, Koricheva J, Noble DWA (2021). Preferred reporting items for systematic reviews and meta-analyses in ecology and evolutionary biology: a PRISMA extension. *Biol Rev Camb Philos Soc*.

[25] Hug LA, Baker BJ, Anantharaman K, Brown CT, Probst AJ (2016). A new view of the tree of life. Nat Microbiol.

[26] Dewar AE, Hao CH, Belcher LJ, Ghoul M, West SA (2024). Bacterial lifestyle shapes pangenomes. Proc Natl Acad Sci USA.

[27] Therneau TM (2024).

[28] Team R.C (2024). R: a language and environment for statistical computing. (ed. Computing R.F.f.S.).

[29] Hadfield JD, Nakagawa S (2010). General quantitative genetic methods for comparative biology: phylogenies, taxonomies and multi-trait models for continuous and categorical characters. J Evol Biol.

[30] Cabeen MT, Jacobs-Wagner C (2005). Bacterial cell shape. Nat Rev Microbiol.

[31] Deng Y, Sun MZ, Shaevitz JW (2011). Direct measurement of cell wall stress stiffening and turgor pressure in live bacterial cells. Phys Rev Lett.

[32] Abt MC, Pamer EG (2014). Commensal bacteria mediated defenses against pathogens. Curr Opin Immunol.

[33] Forterre P, Prangishvili D (2013). The major role of viruses in cellular evolution: facts and hypotheses. Curr Opin Virol.

[34] Kovacs-Simon A, Titball RW, Michell SL (2011). Lipoproteins of bacterial pathogens. Infect Immun.

[35] Zgurskaya HI, Löpez CA, Gnanakaran S (2015). Permeability barrier of Gram-negative cell envelopes and approaches to bypass it. ACS Infect Dis.

[36] Claessen D, Errington J (2019). Cell wall deficiency as a coping strategy for stress. Trends Microbiol.

[37] Ramijan K, Ultee E, Willemse J, Zhang Z, Wondergem JAJ (2018). Stress-induced formation of cell wall-deficient cells in filamentous actinomycetes. Nat Commun.

[38] Chandler CE, Ernst RK (2017). Bacterial lipids: powerful modifiers of the innate immune response. *F1000Res*.

[39] Cross T, Ransegnola B, Shin J-H, Weaver A, Fauntleroy K (2019). Spheroplast-mediated carbapenem tolerance in Gram-negative pathogens. Antimicrob Agents Chemother.

[40] Monahan LG, Turnbull L, Osvath SR, Birch D, Charles IG (2014). Rapid conversion of *Pseudomonas aeruginosa* to a spherical cell morphotype facilitates tolerance to carbapenems and penicillins but increases susceptibility to antimicrobial peptides. Antimicrob Agents Chemother.

[41] Errington J, Mickiewicz K, Kawai Y, Wu LJ (2016). L-form bacteria, chronic diseases and the origins of life. Phil Trans R Soc B.

[42] Innes CMJ, Allan EJ (2001). Induction, growth and antibiotic production of *Streptomyces viridifaciens* L-form bacteria. J Appl Microbiol.

[43] Weller DL, Kovac J, Roof S, Kent DJ, Tokman JI (2017). Survival of *Escherichia coli* on lettuce under field conditions encountered in the northeastern United States. J Food Prot.

[44] Kinnari TJ, Esteban J, Martin-de-Hijas NZ, Sánchez-Muñoz O, Sánchez-Salcedo S (2009). Influence of surface porosity and pH on bacterial adherence to hydroxyapatite and biphasic calcium phosphate bioceramics. J Med Microbiol.

[45] Stine SW, Song I, Choi CY, Gerba CP (2005). Effect of relative humidity on preharvest survival of bacterial and viral pathogens on the surface of cantaloupe, lettuce, and bell peppers. J Food Prot.

[46] Neely AN, Maley MP (2000). Survival of enterococci and staphylococci on hospital fabrics and plastic. J Clin Microbiol.

