# Supplementary material for: Testing the drivers of environmental persistence in bacterial pathogens
Source: Microbiology (Reading). 2026 Jun 8;172(6):001713. doi: 10.1099/mic.0.001713 (PMC13245882; doi:10.1099/mic.0.001713)
Supplement: Supplementary Material 1. [file mic-172-01713-s001.pdf]

Marde et al.

Supplementary Material

Supplementary Methods

The construct the phylogeny used in the work (Figure 1), we followed the methods of Dewar et al. [1], starting with the genus-level 16S phylogeny of Hug et al. [2]. In our data set, 17/29 species were the unique representatives of their genus, and so could be placed immediately on this tree (treating *Chlamydophila* and *Chlamydia*) as synonyms. We did, however, treat *Shigella dysenteriae* as part of the *Escherichia coli* complex, instead of using Hug et al.'s placement of *Shigella flexneri* as basal to a (*Salmonella*, *Escherichia*) pair. To place multiple species in the same genus, we followed the broad procedure of Dewar et al. [3]. In particular, for the genera with two species included our data set (*Neisseria*, *Streptococcus* and the *E. coli*/*Shigella* complex), we placed the split at a quarter of the length of the relevant branch. For the two genera with >2 species (*Acinetobacter* and *Enterococcus*) we followed the topologies described by, respectively, Mateo-Estrada et al. [4] and Schwartzman et al. [5], placing the splits at 1/8, 1/4 and 1/2 of the length of the relevant branch. This is clearly an *ad hoc* method of assigning branch lengths for the youngest nodes. However, we believe it would be sufficient for capturing the major covariances among traits that show phylogenetic signal, while also avoiding singularities when inverting a matrix that includes both multiple phyla and congeners [6]. Finally, we pruned the resulting tree, so that it contained only our 29 species, and reversed the sign of the small magnitude, but negative-valued basal branch.

Supplementary Table S1: List of studies that we excluded from the analysis at full text screening stage.

Study	Reason for exclusion
Ansari et al. (1988)	*Reported on the environmental persistence of non-bacterial pathogens.*
Ansari et al. (1991)	
Bardell (1990)	
Bean et al. (1982)	
Belanov et al. (1996)	
Bond et al. (1981)	
Brady et al. (1990)	
Brown and Gajdusek (1991)	
Buckland and Tyrell (1962)	
Clay et al. (2006)	
DeJong and Winkler (1964)	
Downie and Dumbell (1947)	
Dublineau et al. (2011)	
Edward (1941)	
Firqet et al. (2015)	
Gordon et al. (1993)	
Hall et al. (1980)	
Hara et al. (1990)	
Harper (1961)	
Harper (1963)	
Hendley et al. (1973)	
Karim et al (1985)	
Kingston (1968)	
Kotwal and Cannon (2014)	
Lai et al. (2005)	
Larson and Bryson (1985)	
Lebarbenchon et al. (2011)	
MacCallum and McDonald (1957)	
Mahl and Sadler (1975)	
Mbithi et al. (1991)	
Mbithi et al. (1992)	
Miller and Artenstein (1967)	
Neely and Orloff (2001)	
Nerurkar et al. (1983)	
Paintsil et al. (2014)	
Parker and MacNeal (1944)	
Parker et al. (1944)	
Parkinson et al. (1983)	
Perry et al. (2016)	
Rabenau et al. (2005)	
Rechsteiner and Winkler (1969)	
Reed (1975)	
Rigotto et al. (2011)	
Roden et al. (1997)	
Roger and Faix (1985)	
Satterfield et al. (2017)	
Schoenbaum (1991)	
Stowell et al. (2011)	
Thomas et al. (2008)	
Tiwari et al. (2005)	

Traore et al. (2002)	
van Beuren et al. (1993)	
Wolff and Croon (1968)	
Zarkov and Urumova (2013)	
Walther and Ewald (2004)	*No original persistence data.*
Mitscherlich and Marth (2012)	

Supplementary Table S2: Bacterial taxonomic classification, cell wall structure (gram-positive vs negative), transmission mode (direct vs indirect) and generation time. References are provided for taxonomic classification and generation time.

Bacterial species	Phylum	Class	Order	Ref taxonomy	Cell wall structure	Lifestyle	Ref lifestyle	Transmission mode	Generation time	Ref gen. time
Acinetobacter baumannii	*Pseudomonadota*	*Gammaproteobacteria*	*Pseudomonadales*	[2, 4]	Gram-	Opportunistic	[7]	Indirect	0.5	[8]
Acinetobacter lwoffii	*Pseudomonadota*	*Gammaproteobacteria*	*Pseudomonadales*	[2, 4]	Gram-	Opportunistic	[9]	Indirect	0.5	[8]
Acinetobacter radioresistens	*Pseudomonadota*	*Gammaproteobacteria*	*Pseudomonadales*	[2, 4]	Gram-	Opportunistic	[9]	Indirect	0.5	[8]
Bordetella pertussis	*Pseudomonadota*	*Betaproteobacteria*	*Burkholderiales*	[2]	Gram-	Obligate	[10]	Direct	3.8	[8]
Campylobacter jejuni	*Campylobacterota*	*Campylobacteria*	*Campylobacterales*	[2]	Gram-	Opportunistic	[11]	Indirect	1.1	[8]
Chlamydia pneumoniae	*Chlamydiota*	*Chlamydiia*	*Chlamydiales*	[2, 12]	Gram-	Obligate	[13]	Direct	7.3	[14]
Corynebacterium diphtheriae	*Actinomycetota*	*Actinomycetia*	*Mycobacteriales*	[2]	Gram+	Obligate	[15]	Direct	1.8	[16]
Enterococcus casseliflavus	*Bacillota*	*Bacilli*	*Lactobacillales*	[2, 5]	Gram+	Opportunistic	[17]	Indirect	0.35	[18]
Enterococcus faecalis	*Bacillota*	*Bacilli*	*Lactobacillales*	[2, 5]	Gram+	Opportunistic	[17]	Indirect	0.5	[19]
Enterococcus faecium	*Bacillota*	*Bacilli*	*Lactobacillales*	[2, 5]	Gram+	Opportunistic	[17]	Indirect	1.0	[20]
Enterococcus gallinarum	*Bacillota*	*Bacilli*	*Lactobacillales*	[2, 5]	Gram+	Opportunistic	[17]	Indirect	0.7	[21]
Enterococcus hirae	*Bacillota*	*Bacilli*	*Lactobacillales*	[2, 5]	Gram+	Opportunistic	[17]	Indirect	2.4	[22]
Escherichia coli	*Pseudomonadota*	*Gammaproteobacteria*	*Enterobacterales*	[2, 23]	Gram-	Opportunistic	[24]	Indirect	0.4	[25]
Klebsiella pneumoniae	*Pseudomonadota*	*Gammaproteobacteria*	*Enterobacterales*	[2]	Gram-	Opportunistic	[26]	Direct	3.0	[27]
Listeria monocytogenes	*Bacillota*	*Bacilli*	*Bacillales*	[2]	Gram+	Opportunistic	[28]	Indirect	1.0	[8, 25]
Mycobacterium tuberculosis	*Actinomycetota*	*Actinomycetia*	*Mycobacteriales*	[2]	Gram+	Obligate	[29]	Direct	18.0	[30]

Mycoplasma pneumoniae	*Mycoplasmatota*	*Mollicutes*	*Mycoplasmoidales*	[2]	Gram-	Obligate	[31]	Indirect	30	[32]
Neisseria gonorrhoeae	*Pseudomonadota*	*Betaproteobacteria*	*Neisseriales*	[2, 12]	Gram-	Obligate	[33]	Direct	93.5	[34]
Neisseria meningitidis	*Pseudomonadota*	*Betaproteobacteria*	*Neisseriales*	[2, 12]	Gram-	Obligate	[35]	Direct	0.7	[8, 25]
Proteus mirabilis	*Pseudomonadota*	*Gammaproteobacteria*	*Enterobacterales*	[2]	Gram-	Opportunistic	[36]	Direct	0.5	[37]
Pseudomonas aeruginosa	*Pseudomonadota*	*Gammaproteobacteria*	*Pseudomonadales*	[2]	Gram-	Opportunistic	[38]	Indirect	0.5	[8, 25]
Salmonella enterica serovar Enteritidis	*Pseudomonadota*	*Gammaproteobacteria*	*Enterobacterales*	[2]	Gram-	Obligate	[39]	Indirect	1.1	[40]
Serratia marcescens	*Pseudomonadota*	*Gammaproteobacteria*	*Enterobacterales*	[2]	Gram-	Opportunistic	[41]	Direct	1.0	[42]
Shigella dysenteriae	*Pseudomonadota*	*Gammaproteobacteria*	*Enterobacterales*	[2, 23]	Gram-	Obligate	[43]	Indirect	0.7	[8]
Staphylococcus aureus	*Bacillota*	*Bacilli*	*Bacillales*	[2]	Gram+	Opportunistic	[44]	Direct	0.4	[8, 25]
Streptococcus pneumoniae	*Bacillota*	*Bacilli*	*Lactobacillales*	[2, 12]	Gram+	Opportunistic	[45]	Direct	0.75	[46]
Streptococcus pyogenes	*Bacillota*	*Bacilli*	*Lactobacillales*	[2, 12]	Gram+	Opportunistic	[47]	Direct	0.4	[8, 25]
Vibrio cholerae	*Pseudomonadota*	*Gammaproteobacteria*	*Vibrionales*	[2]	Gram-	Opportunistic	[48]	Indirect	0.2	[8, 25]
Yersinia pestis	*Pseudomonadota*	*Gammaproteobacteria*	*Enterobacterales*	[2]	Gram-	Obligate	[49]	Indirect	1.6	[50]

30

Supplementary Table S3: Surfaces on which bacteria were deposited when measuring their environmental persistence. We provide the surface used and the category to which we allocated it in our analyses (i.e., inorganic or organic).

Surface	Category	Reference
Aerosol	Inorganic	[51-54]
Aluminium	Inorganic	[55]
Asphalt	Inorganic	[56]
Bed Rails	Inorganic	[57]
Brass	Inorganic	[58]
Bronze	Inorganic	[58]
Ceramic	Inorganic	[56]
Concrete	Inorganic	[59]
Copper	Inorganic	[58]
Copper Nickel	Inorganic	[58]
Copper Nickel Zinc	Inorganic	[58]
Countertops	Inorganic	[60]
Detergent	Inorganic	[61]
Drinkers	Inorganic	[59]
Enamel	Inorganic	[62]
Fingertips (Gloved)	Inorganic	[57]
Food Troughs	Inorganic	[59]
Formica	Inorganic	[62, 63]
Glass	Inorganic	[55, 56, 64-73]
Plastic	Inorganic	[55, 74-77]
Polyester	Inorganic	[75, 78, 79]
Polyethylene	Inorganic	[65, 78, 79]
Polyurethane	Inorganic	[78]
Polyvinyl Chloride	Inorganic	[80]
River Water	Inorganic	[81]
Sand	Inorganic	[82]
Soil	Inorganic	[81-83]
Spandex	Inorganic	[78]
SPI-ARGENT II	Inorganic	[84]
Stainless Steel	Inorganic	[56, 58, 62, 65, 80, 81, 85, 86]
Wall	Inorganic	[59]
Water	Inorganic	[61, 83, 87]
Blend	Organic	[78, 79]
Blood Agar	Organic	[88]
Cattle Slurry	Organic	[81]
Cloth	Organic	[55]
Cotton	Organic	[78, 79, 89]
Cotton (Amies Medium)	Organic	[89]
Cotton (Stuart's Medium)	Organic	[89]
Cotton Swab	Organic	[88]
Dry Mops	Organic	[90]
Dust	Organic	[59, 82, 91, 92]
Fabric	Organic	[52, 82]
Faeces	Organic	[59, 81]
Fingertips	Organic	[60]
Linen	Organic	[93]
Linen Sheet	Organic	[76]
Litter	Organic	[59]

Nest Boxes	Organic	[59]
Paper	Organic	[52, 65, 66, 82, 94, 95]
Rubber	Organic	[56, 80]
Sewage	Organic	[87]
Silk	Organic	[56]
Skin	Organic	[63]
Terry	Organic	[78, 79]
Tissue Paper	Organic	[63]
Various Fruits and Vegetables	Organic	[96, 97]
Wood	Organic	[55, 74]

Supplementary Table S5: A list of studies in our data set that could be used in a two-stage meta-analysis, fitting the model to individual studies, before comparing effect sizes. Each study listed both observed mortalities, and tested on both “organic” and “inorganic” surfaces, according to our classification. Unfortunately, there were insufficient sample sizes to make this a practical approach, and the model fitting failed to converge in several cases.

Study	Sample size	Notes
[74]	2	
[87]	4	Two surfaces, each at 2 temperatures
[59]	8	
[93]	3	Temperature varied on one surface only
[63]	3	Failed convergence
[55]	5	Failed convergence
[82]	5	Failed convergence
[81]	5	
[56]	28	
[52]	4	Temperature varied on one surface only; Failed convergence
[65]	8	Failed convergence
[66]	2	Failed convergence

Supplementary Table S6 A list of studies in our data set that scored both gram-negative and gram-positive bacteria. The sample sizes available did not allow for a meaningful two-stage meta-analysis.

Study	Sample size	Gram-positive species	Mortality observed?
[94]	4	*Staphylococcus aureus; Enterococcus hirae*	No
[84]	3	*Staphylococcus aureus*	No
[85]	3	*Staphylococcus aureus*	Yes
[77]	8	*Streptococcus pneumoniae*	No
[62]	9	*Staphylococcus aureus*	Yes

Figure S1: PRISMA flow chart of literature search and selection process for publications of relevance. We list the studies that we excluded from the analysis in Supplementary Table S3. Note that, as all papers were selected by hand, there were no duplicates initially entered into the dataset.

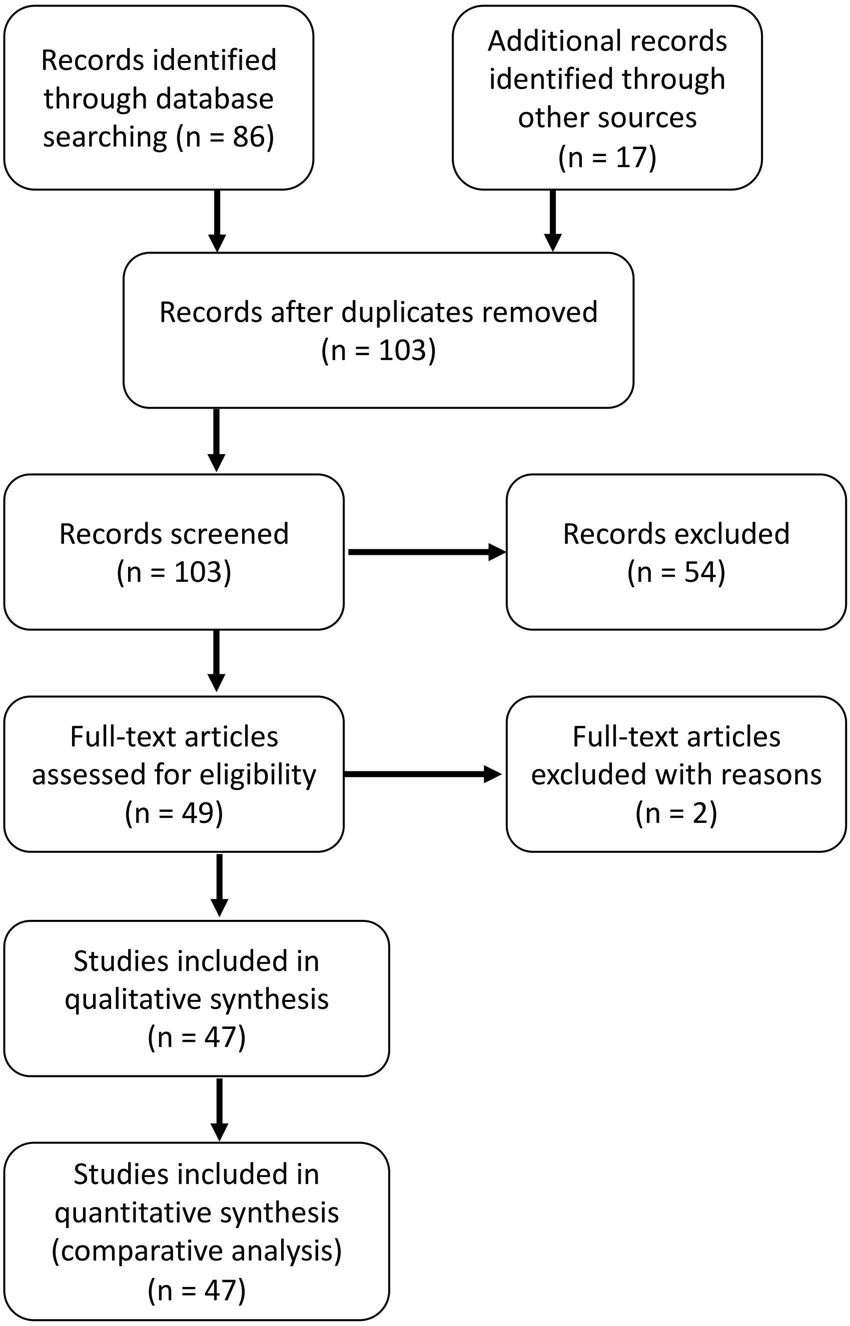

Supplementary Figure S2: Apparent effects of temperature may be due to sampling biases. A) The temperature values in our data set show two clusters at around 20°C and 24°C, and a third smaller cluster of much lower temperatures (<5°C). B) survival times for records where mortality was included suggest. The red empty circles are data points for which humidity was also included. These points are overrepresented at lower temperatures. This explains why results in Table 1 differ for the subset of data with humidity measurements and suggest that the significant effect of temperature might be due entirely to the difference in mean survival times between the two remaining clusters (at ~20°C and 24°C).

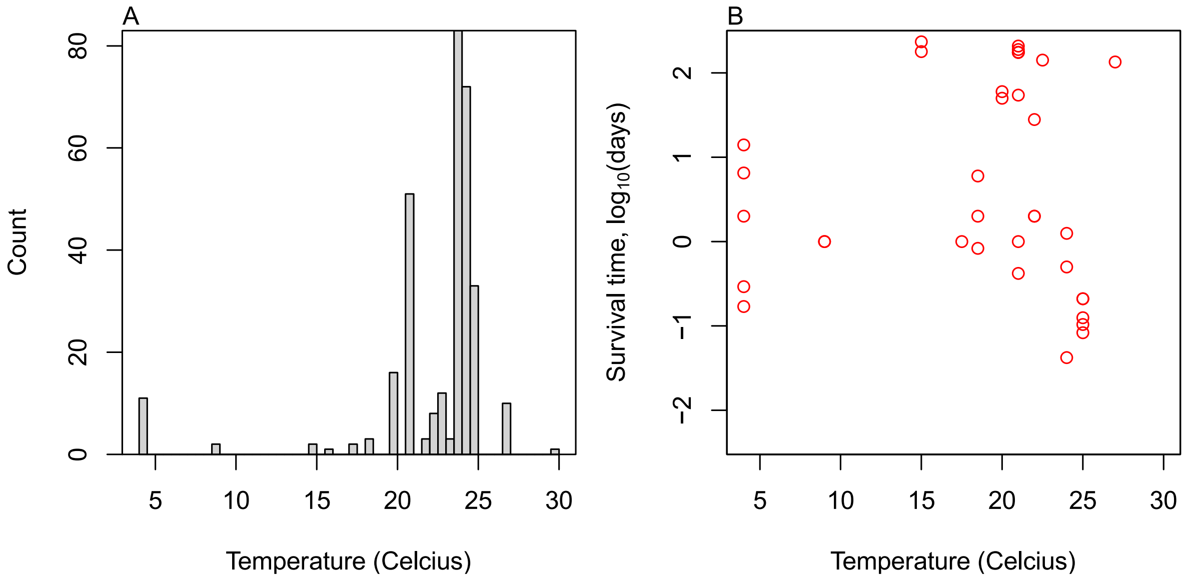

Literature

1. Dewar A.E., Hao C.H., Belcher L.J., Ghoul M., West S.A. 2024 Bacterial lifestyle shapes pangenomes. *Proc Natl Acad Sci U S A* **121**(21), 9. (doi:10.1073/pnas.2320170121).
2. Hug L.A., Baker B.J., Anantharaman K., Brown C.T., Probst A.J., Castelle C.J., Butterfield C.N., Hernsdorf A.W., Amano Y., Ise K., et al. 2016 A new view of the tree of life. *Nat Microbiol* **1**(5), 6. (doi:10.1038/nmicrobiol.2016.48).
3. Dewar A.E., Thomas J.L., Scott T.W., Wild G., Griffin A.S., West S.A., Ghoul M. 2021 Plasmids do not consistently stabilize cooperation across bacteria but may promote broad pathogen host-range. *Nature Ecology & Evolution* **5**(12), 1624-+. (doi:10.1038/s41559-021-01573-2).
4. Mateo-Estrada V., Graña-Miraglia L., López-Leal G., Castillo-Ramírez S. 2019 Phylogenomics reveals clear cases of misclassification and genus-wide phylogenetic markers for Acinetobacter. *Genome Biology and Evolution* **11**(9), 2531-2541. (doi:10.1093/gbe/evz178).
5. Schwartzman J.A., Lebreton F., Salamzade R., Shea T., Martin M.J., Schaufler K., Urhan A., Abeel T., Camargo I., Sgardioli B.F., et al. 2024 Global diversity of enterococci and description of 18 previously unknown species. *Proc Natl Acad Sci U S A* **121**(10), 12. (doi:10.1073/pnas.2310852121).
6. Hadfield J.D., Nakagawa S. 2010 General quantitative genetic methods for comparative biology: phylogenies, taxonomies and multi-trait models for continuous and categorical characters. *J Evol Biol* **23**(3), 494-508. (doi:10.1111/j.1420-9101.2009.01915.x).
7. Howard A., O'Donoghue M., Feeney A., Sleator R.D. 2012 *Acinetobacter baumannii* An emerging opportunistic pathogen. *Virulence* **3**(3), 243-250. (doi:10.4161/viru.19700).
8. Vieira-Silva S., Rocha E.P. 2010 The systemic imprint of growth and its uses in ecological (meta) genomics. *PLoS genetics* **6**(1).
9. Dortet L., Legrand P., Soussy C.J., Cattoir V. 2006 Bacterial identification, clinical significance, and antimicrobial susceptibilities of *Acinetobacter ursingii* and *Acinetobacter schindleri*, two frequently misidentified opportunistic pathogens. *J Clin Microbiol* **44**(12), 4471-4478. (doi:10.1128/jcm.01535-06).
10. Babu M.M., Bhargavi J., Saund R.S., Singh S.K. 2001 Virulence factors of *Bordetella pertussis*. *Curr Sci*, 1512-1522.
11. Tram G., Day C.J., Korolik V. 2020 Bridging the gap: a role for *Campylobacter jejuni* biofilms. *Microorganisms* **8**(3), 11. (doi:10.3390/microorganisms8030452).
12. Dewar A.E., Belcher L.J., West S.A. 2025 A phylogenetic approach to comparative genomics. *Nat Rev Genet* **26**(6), 395-405. (doi:10.1038/s41576-024-00803-0).
13. Huston W.M., Barker C.J., Chacko A., Timms P. 2014 Evolution to a chronic disease niche correlates with increased sensitivity to tryptophan availability for the obligate intracellular bacterium *Chlamydia pneumoniae*. *J Bacteriol* **196**(11), 1915-1924. (doi:10.1128/jb.01476-14).
14. Myers G.S.A., Mathews S.A., Eppinger M., Mitchell C., O'brien K.K., White O.R., Benahmed F., Brunham R.C., Read T.D., Ravel J., et al. 2009 Evidence that human *Chlamydia pneumoniae* was zoonotically acquired. . *J Bacteriol* **191**(23), 7225-7233.
15. Arisoy E.S., Demmler G.J., Dunne W.M. 1993 *Corynebacterium xerosis* ventriculoperitoneal shunt infection in an infant - report of a case and review of the literature. *Pediatr Infect Dis J* **12**(6), 536-538. (doi:10.1097/00006454-199306000-00017).

16. Ott L., Hacker E., Kunert T., Karrington I., Etschel P., Lang R., Wiesmann V., Wittenberg T., Singh A., Varela C., et al. 2017 Analysis of *Corynebacterium diphtheriae* macrophage interaction: Dispensability of corynomycolic acids for inhibition of phagolysosome maturation and identification of a new gene involved in synthesis of the corynomycolic acid layer. *PLoS One* **12**(7), 24. (doi:10.1371/journal.pone.0180105).
17. Krawczyk B., Wityk P., Galecka M., Michalik M. 2021 The many faces of enterococcus spp.-commensal, probiotic and opportunistic pathogen. *Microorganisms* **9**(9), 20. (doi:10.3390/microorganisms9091900).
18. Camargo F.P., Sakamoto I.K., Silva E.L., Duarte I.C.S., Varesche M.B.A. 2021 Bioaugmentation withEnterococcus casseliflavus: A hydrogen-producing strain isolated from citrus peel waste. *Waste and Biomass Valorization* **12**(2), 895-911. (doi:10.1007/s12649-020-01049-7).
19. Salem A.S., Tompkins G.R., Cathro P.R. 2022 Alkaline tolerance and biofilm formation of root canal isolates of *Enterococcus faecalis*: AnIn VitroStudy. *J Endod* **48**(4), 542-+. (doi:10.1016/j.joen.2022.01.006).
20. Morandi S., Brasca M., Alfieri P., Lodi R., Tamburini A. 2005 Influence of pH and temperature on the growth of *Enterococcus faecium* and *Enterococcus faecalis. Lait* **85**(3), 181-192. (doi:10.1051/lait:2005006).
21. Reynolds P.E., Snaith H.A., Maguire A.J., Dutkamalen S., Courvalin P. 1994 Analysis of peptidoglycan precursors in vancomycin-resistant *Enterococcus gallinarum* BM4174. *Biochem J* **301**, 5-8. (doi:10.1042/bj3010005).
22. Belli W.A., Marquis R.E. 1991 Adaptation of *Streptococcus mutans* and *Enterococcus hirae* to acid stress in continuous culture. *Applied and Environmental Microbiology* **57**(4), 1134-1138. (doi:10.1128/aem.57.4.1134-1138.1991).
23. Geurtsen J., de Been M., Weerdenburg E., Zomer A., McNally A., Poolman J. 2022 Genomics and pathotypes of the many faces of *Escherichia coli*. *Fems Microbiol Rev* **46**(6), 30. (doi:10.1093/femsre/fuac031).
24. Walther B., Tedin K., Lübke-Becker A. 2017 Multidrug-resistant opportunistic pathogens challenging veterinary infection control. *Veterinary Microbiology* **200**, 71-78. (doi:10.1016/j.vetmic.2016.05.017).
25. Gama J.A., Abby S.S., Vieira-Silva S., Dionisio F., Rocha E.P.C. 2012 Immune Subversion and Quorum-Sensing Shape the Variation in Infectious Dose among Bacterial Pathogens. *PLoS Pathog* **8**(2), 9. (doi:10.1371/journal.ppat.1002503).
26. Li J., Gao Q.N., Ma Y.Z., Deng Y., Li S.Y., Shi N., Niu H.T., Liu X.Y., Cai J. 2022 Causality of Opportunistic Pathogen *Klebsiella pneumoniae* to Hypertension Development. *Hypertension* **79**(12), 2743-2754. (doi:10.1161/hypertensionaha.122.18878).
27. Schmitz R.A. 2000 Internal glutamine and glutamate pools in *Klebsiella pneumoniae* grown under different conditions of nitrogen availability. *Curr Microbiol* **41**(5), 357-362. (doi:10.1007/s002840010149).
28. Schuppler M., Loessner M.J. 2010 The opportunistic pathogen *Listeria monocytogenes*: pathogenicity and interaction with the mucosal immune system. *International journal of inflammation* **704321**. (doi:doi: 10.4061/2010/704321).
29. Gopinath K., Venclovas C., Ioerger T.R., Sacchettini J.C., McKinney J.D., Mizrahi V., Warner D.F. 2013 A vitamin B12 transporter in *Mycobacterium tuberculosis*. *Open Biol* **3**, 10. (doi:10.1098/rsob.120175).
30. Wayne L.G. 1977 Synchronized replication of *Mycobacterium tuberculosis*. *Infect Immun* **17**(3), 528-530. (doi:10.1128/iai.17.3.528-530.1977).

31. Himmelreich R., Hilbert H., Plagens H., Pirkl E., Li B.C., Herrmann R. 1996 Complete sequence analysis of the genome of the bacterium *Mycoplasma pneumoniae*. *Nucleic Acids Research* **24**(22), 4420-4449. (doi:10.1093/nar/24.22.4420).
32. Halbedel S., Hames C., Stülke J. 2004 In vivo activity of enzymatic and regulatory components of the phosphoenolpyruvate: sugar phosphotransferase system in *Mycoplasma pneumoniae*. *J Bacteriol* **186**(23), 7936-7943. (doi:10.1128/jb.186.23.7936-7943.2004).
33. Tobiason D.M., Seifert H.S. 2006 The obligate human pathogen, *Neisseria gonorrhoeae*, is polyploid. *PLoS Biol* **4**(6), 1069-1078. (doi:10.1371/journal.pbio.0040185).
34. La Scolea Jr L.J., Young F.E. 1974 Development of a defined minimal medium for the growth of *Neisseria gonorrhoeae*. *Applied microbiology* **28**(1), 70-76.
35. Hollingshead S., Tang C.M. 2019 An overview of *Neisseria meningitidis*. In *Neisseria meningitidis: Methods and Protocols* (pp. 1-16.
36. Pellegrino R., Scavone P., Umpiérrez A., Maskell D.J., Zunino P. 2013 Proteus mirabilis uroepithelial cell adhesin (UCA) fimbria plays a role in the colonization of the urinary tract. *Pathog Dis* **67**(2), 104-107. (doi:10.1111/2049-632x.12027).
37. Senior B.W. 1983 *Proteus morgani* is less frequently associated with urinary tract infections than Proteus mirabilis—an explanation. *J Med Microbiol* **16**, 317-322.
38. De Bentzmann S., Plésiat P. 2011 The *Pseudomonas aeruginosa* opportunistic pathogen and human infections. *Environ Microbiol* **13**(7), 1655-1665.
39. Bennett D.D., Higgins S.E., Moore R.W., Beltran R., Caldwell D.J., Byrd J.A., Hargis B.M. 2003 Effects of lime on *Salmonella enteritidis* survival in vitro. *J Appl Poult Res* **12**(1), 65-68. (doi:10.1093/japr/12.1.65).
40. Fehlhaber K., Krüger G. 1998 The study of *Salmonella enteritidis* growth kinetics using rapid automated bacterial impedance technique. *Journal of Applied Microbiology* **84**(6), 945-949.
41. Kurz C.L., Chauvet S., Andrès E., Aurouze M., Vallet I., Michel G.P.F., Uh M., Celli J., Filloux A., de Bentzmann S., et al. 2003 Virulence factors of the human opportunistic pathogen *Serratia marcescens* identified by in vivo screening. *Embo J* **22**(7), 1451-1460. (doi:10.1093/emboj/cdg159).
42. Fedrigo G.V., Campoy E.M., Di Venanzio G., Colombo M.I., Véscovi E.G. 2011*Serratia marcescens* Is Able to Survive and Proliferate in Autophagic-Like Vacuoles inside Non-Phagocytic Cells. *PLoS One* **6**(8). (doi:10.1371/journal.pone.0024054).
43. Moxley R.A. 2022 Enterobacteriaceae: Shigella. *Veterinary Microbiology*, 100-107.
44. O'Gara J.P. 2017 Into the storm: Chasing the opportunistic pathogen *Staphylococcus aureus* from skin colonisation to life-threatening infections. *Environ Microbiol* **19**(10), 3823-3833. (doi:10.1111/1462-2920.13833).
45. Keller L.E., Rueff A.S., Kurushima J., Veening J.W. 2019 Three New Integration Vectors and Fluorescent Proteins for Use in the Opportunistic Human Pathogen *Streptococcus pneumoniae*. *Genes* **10**(5), 15. (doi:10.3390/genes10050394).
46. Paik J., Kern I., Lurz R., Hakenbeck R. 1999 Mutational analysis of the *Streptococcus pneumoniae* bimodular class a penicillin-binding proteins. *J Bacteriol* **181**(12), 3852-3856. (doi:10.1128/jb.181.12.3852-3856.1999).
47. Zhi Y., Chen X.Y., Cao G.X., Chen F.J., Seo H.S., Li F. 2022 The effects of air pollutants exposure on the transmission and severity of invasive infection caused by an opportunistic pathogen *Streptococcus pyogenes*. *Environ Pollut* **310**, 14. (doi:10.1016/j.envpol.2022.119826).

48. Matz C., McDougald D., Moreno A.M., Yung P.Y., Yildiz F.H., Kjelleberg S. 2005 Biofilm formation and phenotypic variation enhance predation-driven persistence of *Vibrio cholerae*. *Proc Natl Acad Sci U S A* **102**(46), 16819-16824. (doi:10.1073/pnas.0505350102).
49. Titball R.W., Hill J., Lawton D.G., Brown K.A. 2003 *Yersinia pestis* and plague. *Biochem Soc Trans* **31**, 104-107. (doi:10.1042/bst0310104).
50. Heine H.S., Chuvala L., Riggins R., Hurteau G., Cirz R., Cass R., Louie A., Drusano G.L. 2013 Natural History of *Yersinia pestis* Pneumonia in Aerosol-Challenged BALB/c Mice. *Antimicrobial Agents and Chemotherapy* **57**(5), 2010-2015. (doi:10.1128/aac.02504-12).
51. Dunklin E.W., Puck T.T. 1948 The lethal effect of relative humidity on air-borne bacteria. *J Exp Med* **87**(2), 87-101. (doi:10.1084/jem.87.2.87).
52. Ocklitz H.W., Milleck J. 1967 Die Uberlebensheit von pertussis bakterien ausserhalb das kranken. Experimentelle untersuchungen zur keuchhustensepidemiologie. *Zentralblatt Bakteriologie Parasitenkunde Infektionskrankheiten* **203**, 79-91.
53. Wells W.F., Stone W.R. 1934 On air-borne infection - Study III Viability of droplet nuclei infection. *American Journal of Hygiene* **20**(3), 619-627. (doi:10.1093/oxfordjournals.aje.a118098).
54. Wright D.N., Bailey G.D., Hatch M.T. 1968 Role of relative humidity in the survival of airborne *Mycoplasma pneumoniae*. *Journal of Bacteriology,* **96**(4), 970-974.
55. Islam M.S., Hossain M.A., Khan S.I., Khan M.N.H., Sack R.B., Albert M.J., Huq A., Colwell R.R. 2001 Survival of *Shigella dysenteriae* type 1 on fomites. *Journal of Health Population and Nutrition* **19**(3), 177-182.
56. McDade J.J., Hall L.B. 1963 Survival of *Staphylococcus aureus* in the Environment. I. Exposure on Surfaces. *American journal of hygiene* **78**(3), 330-337.
57. Catalano M., Quelle L.S., Jeric P.E., Di Martino A., Maimone S.M. 1999 Survival of *Acinetobacter baumannii* on bed rails during an outbreak and during sporadic cases. *Journal of Hospital Infection* **42**(1), 27-35. (doi:10.1053/jhin.1998.0535).
58. Wilks S.A., Michels H., Keevil C.W. 2005 The survival of *Escherichia coli* O157 on a range of metal surfaces. *International Journal of Food Microbiology* **105**(3), 445-454. (doi:10.1016/j.ijfoodmicro.2005.04.021).
59. Davies R.H., Wray C. 1996 Persistence of *Salmonella enteritidis* in poultry units and poultry food. *Br Poult Sci* **37**(3), 589-596. (doi:10.1080/00071669608417889).
60. Noskin G.A., Stosor V., Cooper I., Peterson L.R. 1995 Recovery of vancomycin-resistant enterococci on fingertips and environmental, surfaces. *Infection Control and Hospital Epidemiology* **16**(10), 577-581.
61. Beadle I.R., Verran J. 1999 The survival and growth of an environmental Klebsiella isolate in detergent solutions. *Journal of Applied Microbiology* **87**(5), 764-769. (doi:10.1046/j.1365-2672.1999.00923.x).
62. Webster C., Towner K.J., Humphreys H. 2000 Survival of Acinetobacter on three clinically related inanimate surfaces. *Infection Control and Hospital Epidemiology* **21**(4), 246-246. (doi:10.1086/503214).
63. Falsey A.R., Walsh E.E. 1993 Transmission of *Chlamydia pneumoniae*. *J Infect Dis* **168**(2), 493-496. (doi:10.1093/infdis/168.2.493).
64. Jawad A., Snelling A.M., Heritage J., Hawkey P.M. 1998 Exceptional desiccation tolerance of *Acinetobacter radioresistens*. *Journal of Hospital Infection* **39**(3), 235-240. (doi:10.1016/s0195-6701(98)90263-8).
65. Rose L.J., Donlan R., Banerjee S.N., Arduino M.J. 2003 Survival of *Yersinia pestis* on environmental surfaces. *Applied and Environmental Microbiology* **69**(4), 2166-2171. (doi:10.1128/aem.69.4.2166-2171.2003).

66. Smith C.R. 1942 Survival of Tubercle Bacilli in Books: How Contaminated Books May Be Rendered Noninfectious. *American Review of Tuberculosis* **46**(5), 549-559.
67. Soparkar M.B. 1917 The Vitality of the Tubercle Bacillus outside the Body. *Indian Journal of Medical Research* **4**(4).
68. Wagenvoort J.H.T., De Brauwer E., Penders R.J.R., Willems R.J., Top J., Bonten M.J. 2011 Environmental survival of vancomycin-resistant *Enterococcus faecium*. *Journal of Hospital Infection* **77**(3), 282-283. (doi:10.1016/j.jhin.2010.11.008).
69. Wagenvoort J.H.T., Joosten E. 2002 An outbreak *Acinetobacter baumannii* that mimics MRSA in its environmental longevity. *Journal of Hospital Infection* **52**(3), 226-227. (doi:10.1053/jhin.2001.1294).
70. Wagenvoort J.H.T., Penders R.J.R. 1997 Long-term in-vitro survival of an epidemic MRSA phage-group III-29 strain. *Journal of Hospital Infection* **35**(4), 322-325. (doi:10.1016/s0195-6701(97)90229-2).
71. Wagenvoort J.H.T., Penders R.J.R., Davies B.I., Lütticken R. 2005 Similar environmental survival patterns of *Streptococcus pyogenes* strains of different epidemiologic backgrounds and clinical severity. *European Journal of Clinical Microbiology & Infectious Diseases* **24**(1), 65-67. (doi:10.1007/s10096-004-1256-8).
72. Wagenvoort J.H.T., Sluijsmans W., Penders R.J.R. 2000 Better environmental survival of outbreak vs. sporadic MRSA isolates. *Journal of Hospital Infection* **45**(3), 231-234. (doi:10.1053/jhin.2000.0757).
73. Williams S.G., Kauffman C.A. 1978 Survival of *Streptococcus pneumoniae* in sputum from patients with pneumonia. *J Clin Microbiol* **7**(1), 3-5.
74. Abrishami S.H., Tall B.D., Bruursema T.J., Epstein P.S., Shah D.B. 1994 Bacterial adherence and viability on cutting board surfaces. *Journal of Food Safety* **14**(2), 153-172. (doi:10.1111/j.1745-4565.1994.tb00591.x).
75. Huang R., Mehta S., Weed D., Price C.S. 2006 Methicillin-resistant *Staphylococcus aureus* survival on hospital fomites. *Infection Control and Hospital Epidemiology* **27**(11), 1267-1269. (doi:10.1086/507965).
76. Perez J.L., Gomez E., Sauca G. 1990 Survival of gonococci from urethral discharge on fomites. *European Journal of Clinical Microbiology & Infectious Diseases* **9**(1), 54-55.
77. Tzeng Y.L., Martin L.E., Stephens D.S. 2014 Environmental survival of *Neisseria meningitidis*. *Epidemiology and Infection* **142**(1), 187-190. (doi:10.1017/s095026881300085x).
78. Neely A.N. 2000 A survey of Gram-negative bacteria survival on hospital fabrics and plastics. *Journal of Burn Care & Rehabilitation* **21**(6), 523-527. (doi:10.1097/00004630-200021060-00009).
79. Neely A.N., Maley M.P. 2000 Survival of enterococci and staphylococci on hospital fabrics and plastic. *J Clin Microbiol* **38**(2), 724-726. (doi:10.1128/jcm.38.2.724-726.2000).
80. Wendt C., Dietze B., Dietz E., Ruden H. 1997 Survival of *Acinetobacter baumannii* on dry surfaces. *J Clin Microbiol* **35**(6), 1394-1397. (doi:10.1128/jcm.35.6.1394-1397.1997).
81. Maule A. 2000 Survival of verocytotoxigenic *Escherichia coli* O157 in soil, water and on surfaces. *Journal of Applied Microbiology* **88**, 71S-78S. (doi:10.1111/j.1365-2672.2000.tb05334.x).
82. Laurell G., Lofstrom G., Magnusson J.H., Ouchterlony O. 1949 Airborne infections .2. a report on the methods. *Acta Medica Scandinavica* **134**(3), 189-204.
83. Duffitt A.D., Reber R.T., Whipple A., Chauret C. 2011 Gene expression during survival of *Escherichia coli* O157: H7 in soil and water. *International Journal of Microbiology* **340506**. (doi:10.1155/2011/340506).

84. Kampf G., Dietze B., Grosse-Siestrup C., Wendt C., Martiny H. 1998 Microbicidal activity of a new silver-containing polymer, SPI-ARGENT II. *Antimicrobial Agents and Chemotherapy* **42**(9), 2440-2442. (doi:10.1128/aac.42.9.2440).
85. Kusumaningrum H.D., Riboldi G., Hazeleger W.C., Beumer R.R. 2003 Survival of foodborne pathogens on stainless steel surfaces and cross-contamination to foods. *International Journal of Food Microbiology* **85**(3), 227-236. (doi:10.1016/s0168-1605(02)00540-8).
86. Morita Y., Komoda E., Ono K., Kumagai S. 2011 Survival of Biofilm-Forming Salmonella on Stainless Steel Bolt Threads under Dry Conditions. *Food Hygiene and Safety Science* **52**(5), 299-303. (doi:10.3358/shokueishi.52.299).
87. Budzinska K., Wronski G., Szejniuk B. 2012 Survival Time of Bacteria *Listeria monocytogenes* in Water Environment and Sewage. *Polish Journal of Environmental Studies* **21**(1), 31-37.
88. Downie A.W. 1940 Survival of *Meningoeocei* on Swabs and Blood Agar. *Lancet*, 36-37.
89. Hunter P.R. 1986 Survival of *Bordetella pertussis* in transport media. *Journal of Clinical Pathology* **39**(1), 119-120. (doi:10.1136/jcp.39.1.119).
90. Oie S., Kamiya A. 1996 Survival of methicillin-resistant *Staphylococcus aureus* (MRSA) on naturally contaminated dry mops. *Journal of Hospital Infection* **34**(2), 145-149. (doi:10.1016/s0195-6701(96)90140-1).
91. Crosbie W.E., Wright H.D. 1941 *Diphtheria bacilli* in floor dust. *Lancet* **1**, 656-659.
92. Engley F.B. 1955 The persistence (durability) of microorganisms: I. airborne organisms. *Texas Reports of Biological Medicine* **13**, 712-757.
93. Elmros T. 1977 Survival of *Neisseria gonorrhoeae* on surfaces. *Acta Dermato-Venereologica* **57**(2), 177-180.
94. Hübner N.O., Hübner C., Kramer A., Assadian O. 2011 Survival of bacterial pathogens on paper and bacterial retrieval from paper to hands: preliminary results. *AJN The American Journal of Nursing* **111**(12), 30-34.
95. Kenwood H., Dove E. 1915 The risks from tubercuolus infection retained in books. *The Lancet* **186**(4793), 66-68.
96. Felsenfeld O. 1965 Notes on food beverages and fomites contaminated with vibrio cholerae. *Bulletin of the World Health Organization* **33**(5), 725-+.
97. Weller D.L., Kovac J., Roof S., Kent D.J., Tokman J.I., Kowalcyk B., Oryang D., Ivanek R., Aceituno A., Sroka C., et al. 2017 Survival of *Escherichia coli* on Lettuce under Field Conditions Encountered in the Northeastern United States. *Journal of Food Protection* **80**(7), 1214-1221. (doi:10.4315/0362-028x.Jfp-16-419).
